# Enterovirus 71 Induces Mitochondrial Reactive Oxygen Species Generation That is Required for Efficient Replication

**DOI:** 10.1371/journal.pone.0113234

**Published:** 2014-11-17

**Authors:** Mei-Ling Cheng, Shiue-Fen Weng, Chih-Hao Kuo, Hung-Yao Ho

**Affiliations:** 1 Department of Biomedical Sciences, College of Medicine, Chang Gung University, Tao-Yuan, Taiwan; 2 Healthy Aging Research Center, Chang Gung University, Tao-Yuan, Taiwan; 3 Metabolomics Core Laboratory, Chang Gung University, Tao-Yuan, Taiwan; 4 Department of Medical Biotechnology and Laboratory Science, College of Medicine, Chang Gung University, Tao-Yuan, Taiwan; 5 Office of Research and Development, Chang Gung Memorial Hospital, Tao-Yuan, Taiwan; Chang-Gung University, Taiwan

## Abstract

Redox homeostasis is an important host factor determining the outcome of infectious disease. Enterovirus 71 (EV71) infection has become an important endemic disease in Southeast Asia and China. We have previously shown that oxidative stress promotes viral replication, and progeny virus induces oxidative stress in host cells. The detailed mechanism for reactive oxygen species (ROS) generation in infected cells remains elusive. In the current study, we demonstrate that mitochondria were a major ROS source in EV71-infected cells. Mitochondria in productively infected cells underwent morphologic changes and exhibited functional anomalies, such as a decrease in mitochondrial electrochemical potential ΔΨ_m_ and an increase in oligomycin-insensitive oxygen consumption. Respiratory control ratio of mitochondria from infected cells was significantly lower than that of normal cells. The total adenine nucleotide pool and ATP content of EV71-infected cells significantly diminished. However, there appeared to be a compensatory increase in mitochondrial mass. Treatment with mito-TEMPO reduced eIF2α phosphorylation and viral replication, suggesting that mitochondrial ROS act to promote viral replication. It is plausible that EV71 infection induces mitochondrial ROS generation, which is essential to viral replication, at the sacrifice of efficient energy production, and that infected cells up-regulate biogenesis of mitochondria to compensate for their functional defect.

## Introduction

Enterovirus 71 (EV71), a member of the family *Picornaviridae*, is a non-enveloped RNA virus [1]. Since its identification in 1969 [2], episodes of EV71 outbreak occurred periodically throughout the world [3]–[6]. Clinical manifestation of EV71 infection includes febrile illness, acute respiratory disease, hand-foot-and-mouth disease, herpangia, myocarditis, aseptic meningitis, acute flaccid paralysis, brainstem and/or cerebellar encephalitis, Guillain-Barre syndrome, or combinations of these clinical features [7], [8]. Though hand-foot-and-mouth disease is a benign disease, the neurologic and systemic complications are more severe [9]. Children aged below 5 years are susceptible to development of permanent neurologic sequelae, or even succumb to such complications [10]. To date, the largest epidemic of EV71 occurred in China in 2008 and 2009. Nearly 490000 cases, of which 126 deaths occurred, were reported; over a million cases of hand-foot-and-mouth disease were reported in 2009 [4], [5]. EV71 infection recurs every 2 or 3 years in Asia-Pacific region.

It is evident that redox environment is a factor affecting host-microbe interactions. The susceptibility of host cells to HIV, coxsackievirus and influenza virus is affected by redox microenvironment [11]–[15]. We have recently found that increased oxidative stress in host cells enhances EV71 infection [16]. It is intriguing that viral infection can itself induce oxidative stress in host cells. Increased superoxide production has been observed in pneumonia caused by influenza A virus [17]. Herpes simplex virus (HSV) infection of microglia cells elicits oxidative stress, which probably causes the neurotoxicity [18]. Rhinovirus and respiratory syncytial virus induce ROS production in fibroblasts and epithelial cells [19], [20]. We have recently found that EV71 infection can lead to increased reactive oxygen species (ROS) generation [16]. However, the mechanism for ROS production in EV71-infected cells remains elusive.

Mitochondria are important organelle for cellular energy metabolism, and have been recently implicated in responses of host cell to viral infection. They lie at the crossroad of metabolism, redox homeostasis, apoptosis, and innate immune signaling, and probably form a multi-functional signaling platform termed mitoxosome [21]. Mitochondria-associated adaptors such as MAVS are involved in modulation of innate immunity [22]. Additionally, intermembrane space proteins, such as cytochrome c and Smac/DIABLO, and outer mitochondrial membrane proteins, such as Bcl-2, are involved in initiation and regulation of apoptosis [23], [24]. Viral infection is associated with changes in mitochondrial functions. Hepatitis C virus (HCV)-infected cells have impaired oxidative phosphorylation and increased ROS generation [25], [26]. Expression of HCV protein represses mitochondrial membrane potential and mitochondrial coupling efficiency, and leads to increased ROS production [27]. Likewise, expression of human immunodeficiency virus (HIV) Tat protein, hepatitis B virus X protein, severe acute respiratory syndrome (SARS) coronavirus non-structural protein 10 results in mitochondrial depolarization [28]–[30]. These proteins are known to induce oxidative stress [30]–[32]. It is currently unknown whether EV71-induced oxidative stress is associated with mitochondrial dysfunction.

In the present study, we investigated the mechanistic aspect of ROS generation induced by EV71 infection. We examined the changes in mitochondria in EV71-infected cells. Our findings show that EV71 infection induces mitochondrial dysfunction and anomalous changes in their morphology and subcellular distribution. These mitochondria represent the sites of ROS generation. Such changes are associated with altered cellular energy metabolism. Mitochondrial ROS are essential to viral replication process. The increase in mitochondrial mass in infected cells may compensate for energy deficit caused by abnormal mitochondria.

## Materials and Methods

### Cell culture and virological techniques

SF268 glioblastoma cells (National Cancer Institute Center for Cancer Research ID: 59) were cultured in Dulbecco’s modified Eagle’s medium (DMEM) supplemented with 10% fetal calf serum (FCS), 2 mM glutamine, 2 mM non-essential amino acids, 100 U/ml penicillin, 0.1 mg/ml streptomycin, and 0.25 µg/ml amphotericin at 37°C in a humidified atmosphere of 5% CO_2_ [33], [34]. EV71 (BrCr strain; ATCC VR784) was propagated in Vero cells as described previously [16]. Vero cells (ATCC CCL-81) were cultured in modified Eagle’s medium (MEM) supplemented with 10% FCS, 100 U/ml penicillin, 0.1 mg/ml streptomycin and 0.25 µg/ml amphotericin in an atmosphere of 5% CO_2_ at 37°C. Quantitative PCR analysis of the copy number of EV71 genomic DNA was performed as described previously [16].

### Detection of ROS and ΔΨ_m_ by confocal microscopy

Visualization of ROS generation was performed by confocal microscopy as described previously [35]. The fluorescence of dichlorofluorescein (DCF) is derived from oxidation of its fluorogenic precursor [35]. In brief, cells were grown in glass-bottomed culture dish, and infected with virus at m.o.i. of 1.25. The infected cells were stained with 100 nM MitoTracker Red and 5 µM H_2_DCFDA for 20 min at 37°C. and counterstained with with 5 µg/ml of Hoechst 33342. The specimens were examined with Zeiss LSM 510 Meta system (Carl Zeiss MicroImaging GmbH, Heidelberg, Germany). Confocal fluorescence images of labeled cells were acquired using Plan-Apochromat 100×1.40 NA oil immersion objective. To scan for DCF signal, we used the 488 nm excitation line of argon laser, beam splitter (HFT 405/488/561/633/KP720), and an emission window set at 505–550 nm. To scan for MitoTracker Red signal, we used the 561 nm excitation line of DPSS laser and an emission window set at 575–615 nm. For scanning of Hoechst dye, we used 405 nm excitation line of diode laser and an emission an emission window set at 420–480 nm. To scan for Hoechst dye signal, we used the 405 nm excitation line of diode laser and an emission an emission window set at 420–480 nm. We analyzed all images using Zeiss Zen software package (Carl Zeiss MicroImaging GmbH, Heidelberg, Germany).

Confocal microscopic analysis of ΔΨ_m_ was performed as described previously [36]. The mitochondrial membrane potential was determined using the cationic, lipophilic dye JC-1 (5,5′,6,6′-tetrachloro-1,1′,3,3′-tetraethylbenz- imidazolocarbocyanine iodide) (Invitrogen, CA, USA). JC-1 monomers emit at 527 nm, and J-aggregates emit at 590 nm. Infected cells were loaded with 0.5 µM of JC-1 in culture medium at 37°C for 60 min, and counterstained with 5 µg/ml of Hoechst 33342 prior to examination with Leica TCS SP2 MP system (Leica Microsystems, Mannheim, Germany). Confocal fluorescence images of JC-1 labeled cells were obtained using HCX PL-APO CS 100×1.40 NA oil immersion objective. To scan for signal of JC-1 monomeric form, we used the 488 nm excitation line of argon laser, triple-dichroic beam splitter (TD 488/543/633) and an emission window set at 500–540 nm. To scan for signal of JC-1 aggregated form, we used the 543 nm excitation line of He-Ne laser, triple-dichroic beam splitter (TD 488/543/633) and an emission window set at 580–620 nm. To scan for Hoechst dye signal, we employed a fiber coupling system equipped with a Ti:Sapphire laser (model Millenia/Tsunami; Spectra-Physics; Mountain View, CA, USA), and selected the wavelength of 800 nm for illumination. Image analysis was performed with Leica LCS software packages.

### Cytometric analysis of ROS, mitochondrial mass, and ΔΨ_m_

ROS formation was analyzed quantitatively by cytometric analysis. In brief, infected cells were loaded with 2 µM MitoSOX red or 5 µM H_2_DCFDA for 20 min at 37°C, washed twice with PBS, and trypsinized for flow cytometric analysis as previously described [37]. The mean fluorescence intensity (MFI) of the fluorescence of oxidized MitoSOX red or MFI of DCF fluorescence was quantified using CellQuest Pro software (Becton Dickinson, CA, USA). Likewise, for determination of mitochondrial mass, cells were stained with 100 nM Mitotracker Red 580FM (Invitrogen, CA, USA), and analyzed according to manufacturer’s instruction. MFI was quantified using CellQuest Pro software. For analysis of ΔΨ_m_, infected cells were loaded with 0.5 µM JC-1 as described above. The cell monolayer was washed three times with PBS, and trypsinized for cytometric analysis. Cells were analyzed for JC-1 monomer fluorescence (FL1 channel) and J-aggregate fluorescence (FL2 channel) with CELLQuest software. The ratio of the mean fluorescence intensity of FL2 channel to that of FL1 channel was calculated.

### Measurement of oxygen consumption

SF268 cells were infected with EV71 at m.o.i. of 1.25 for indicated times, washed twice with PBS, and trypsinized. The cells were pelleted, and then resuspended in respiration buffer (20 mM NaKPO4 (pH 7.2), 65 mM KCl, 125 mM sucrose, 2 mM MgCl_2_). The cell suspension was transferred to the respiratory chamber of Mitocell equipped with Clark-type electrode, which was connected to Strathkelvin 928 6-Channel Oxygen System (Strathkelvin Instruments, Glasgow, UK). Oxygen consumption rate was monitored. In some experiments, oligomycin was added to cell suspension at 2 µg/ml. Data are expressed as µg O_2_ per 5×10^5^ cells per min. For data normalization with respect to mitochondrial mass, the oxygen consumption rate was normalized to mitochondrial mass determined by Mitotracker Red 580FM Staining as described above. Data are expressed as µg O_2_ per mitochondrial mass unit (MMU) per min. A similar approach for determination of oxygen consumption rate per unit of mitochondrial mass was previously described [38].

### Isolation of mitochondria and measurement of respiratory control ratio (RCR)

Cells were infected with EV71 at m.o.i. of 1.25, washed twice with PBS, and scrapped in ice-cold SHE buffer (0.25 M sucrose, 1 mM EGTA, 3 mM HEPES). The cells were homogenized. The homogenate was centrifuged at 1000×g at 4°C for 10 min, and subsequently centrifuged at 10000×g at 4°C for 10 min. Finally, the mitochondrial pellet was suspended in a suitable volume of SHE buffer.

For measurement of oxygen consumption of mitochondrial preparation, 0.5 mg of mitochondria was added to 300 µl of assay buffer (20 mM K_2_HPO_4_/KH_2_PO_4_ (pH 7.2), 125 mM sucrose, 65 mM KCl, 2 mM MgCl_2_), and transferred to the Mitocell chamber equipped with Clark-type electrode. Malate and glutamate were added to chamber at the final concentration of 5 mM. ADP was added at a concentration of 0.5 mM to stimulate oxygen consumption. The respiratory control ratio (RCR) is defined as rate of ADP-stimulated state 3 oxygen consumption divided by the rate of oxygen consumption (State 4_o_) determined in the presence of 2 µg/ml oligomycin [39]. The state 4o is a specific indicator of mitochondrial proton movement being limited by leakiness of inner mitochondrial membrane to proton. RCR was calculated as state 3 oxygen consumption rate divided by that of state 4_o_.

### Transmission electron microscopy

The transmission electron microscopy was performed as previously described with slight modifications [40]. Briefly stated, the infected cells were washed three times with PBS; trypsinized; placed in a BEEM capsule; and centrifuged to form a pellet with a size of 0.5–1 mm^3^. The pellet was fixed with fresh 2.5% glutaraldehyde/PBS at 4°C for 2 hr. After fixation, it was rinsed with PBS thrice for 10 min each. One ml of 1% OsO_4_ was added to the cell pellet for 1 hr. The pellet was washed with PBS thrice for 10 min each, and then with 50% ethanol thrice for 10 min each. The specimen was then stained *en bloc* with 0.5% uranyl acetate/50% ethanol at 4°C overnight. It was dehydrated in ethanol with graded concentrations (70%, 80%, 90%, 95%, 100%, 100%, 100%, 100%) for 15 min each. The specimen was successively infiltrated with ethanol/Spurr’s resin (50%∶50%), ethanol/Spurr’s resin (25%∶75%), and pure Spurr’s resin overnight for each treatment. It was then heated at 70°C for 72 hr. Thin sections with thickness of 70 nm were obtained with the use of ultramicrotome, and picked on TEM grids. Sections were stained with 2% uranyl acetate for 30 min, followed by 6 min staining with 0.4% lead citrate. The sections were rinsed with water, blotted dry, and examined on a JEOL JEM-1230 electron microscope.

### Determination of adenine nucleotides

Levels of ATP, ADP and AMP were determined using UPLC under conditions as previously described for measurement of nicotinamide nucleotides [41].

### Western blotting and silver staining

SDS-PAGE and western blotting were performed as previously described [42]. The chemiluminescent signal was detected with X-ray film (Fujifilm, Japan). The silver staining was performed as previously described [43].

## Results

### EV71 induces mitochondrial ROS generation

**Figure 1:**
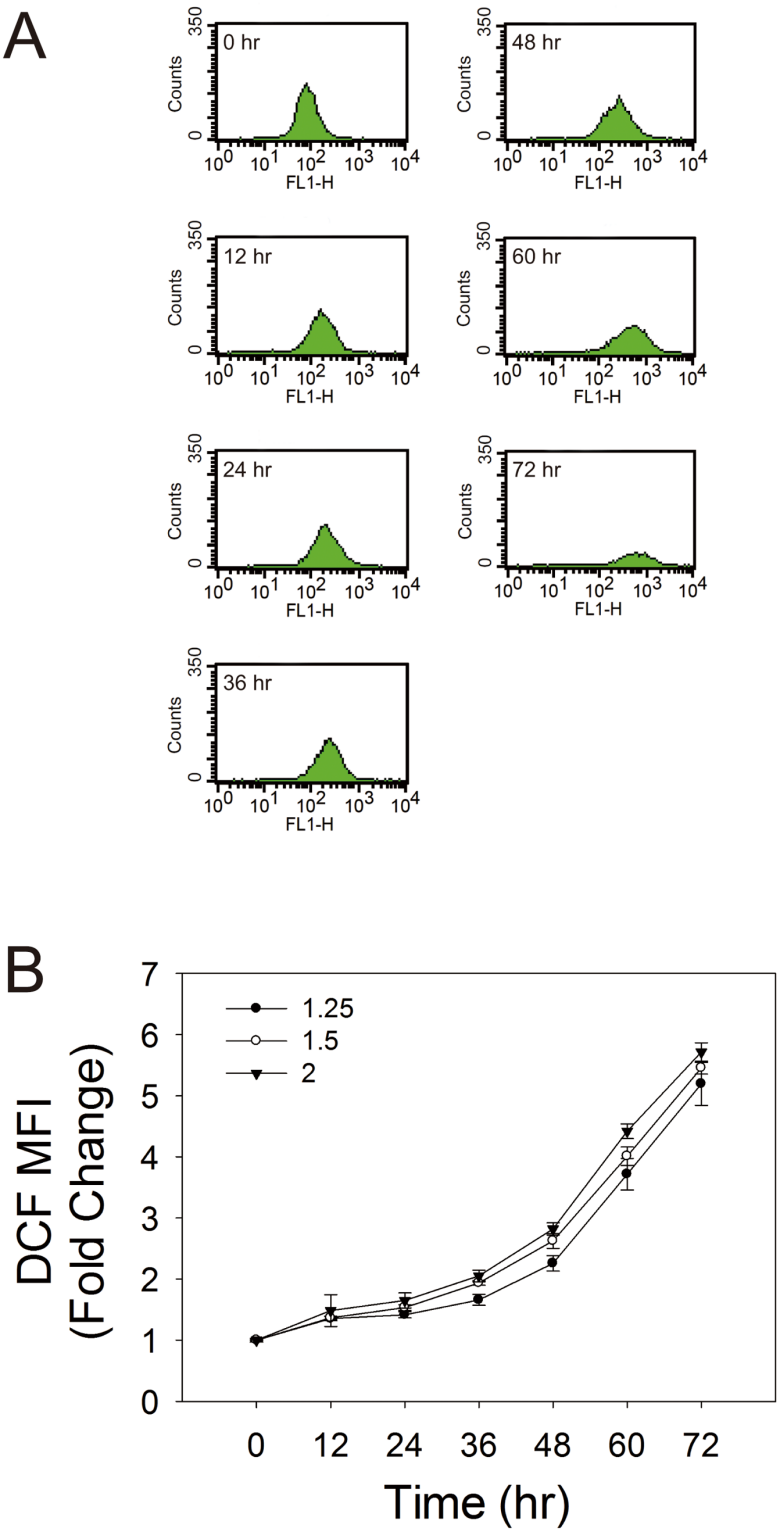
EV71 infection induces ROS in neural cells in a time-dependent manner. SF268 cells were infected with EV71 at m.o.i. of 1.25, 1.5 and 2 for 0, 12, 24, 36, 48, 60 and 72 hr, and were subject to H_2_DCFDA staining and flow cytometric analysis. (A) Representative histograms of cell counts (*counts*) vs. DCF fluorescence (*FL1-H*) for cells infected at an m.o.i. of 1.25 at indicated times are shown. (B) The mean fluorescence intensity (MFI) of DCF of infected cells is expressed as fold change relative to that of uninfected cells. The results are presented as mean±SD of three separate experiments.

**Figure 2:**
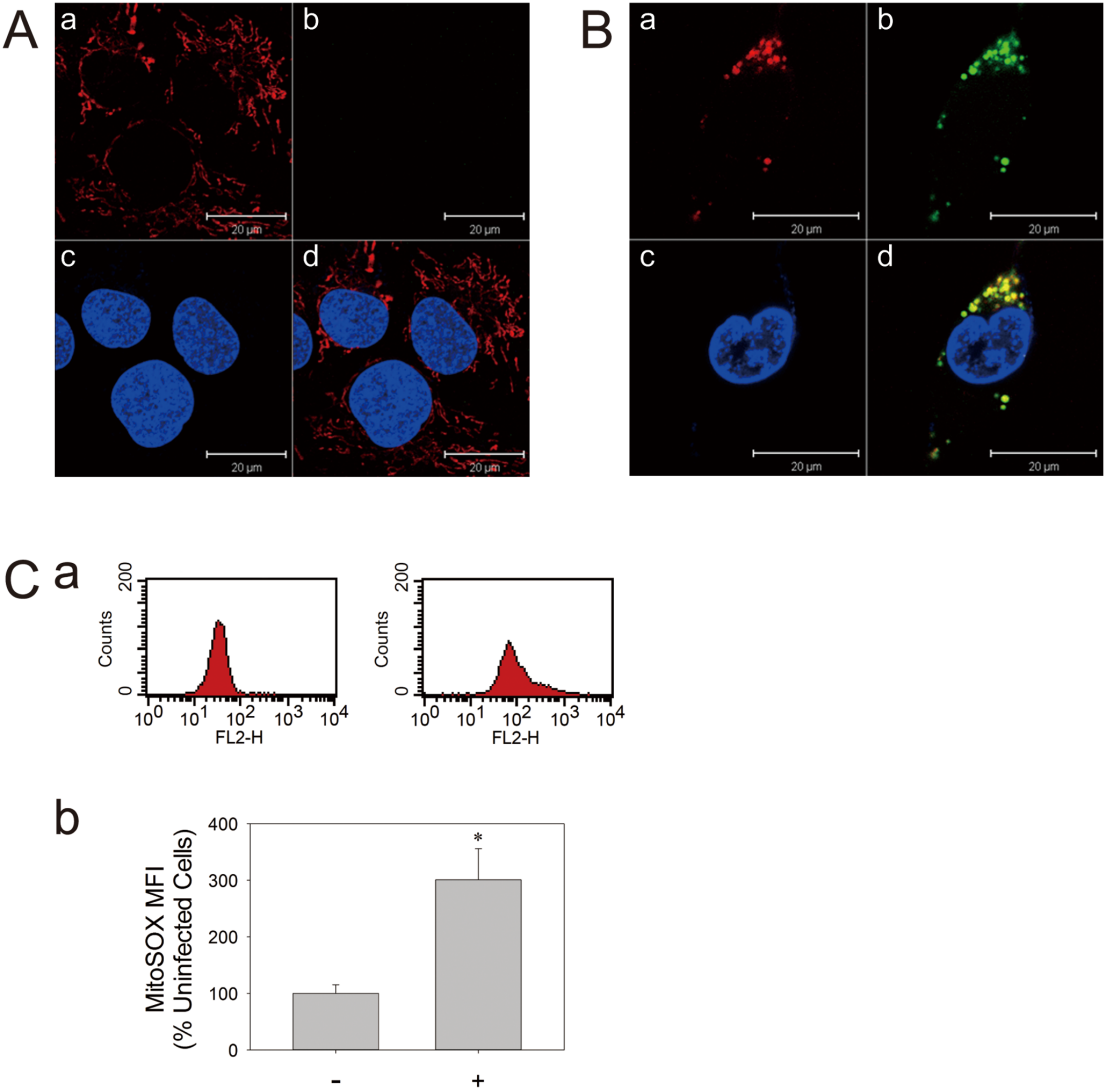
EV71 infection causes mitochondrial ROS production. (A) SF268 cells were mock- (A) or infected with (B) EV71 at an m.o.i. of 1.25 for 54 hr, and were subject to H_2_DCFDA and Mitotracker Red staining and confocal microscopic examination. The MitoSOX-stained mitochondria (a), DCF-stained ROS generation sites (b), and Hoechst 33342-stained nuclei (c) are shown. The corresponding images are overlaid (d). The photographs shown here are representative of three experiments. Scale bar, 20 µm. (C) SF268 cells were mock- or infected with EV71 at an m.o.i. of 1.25 for 48 hr, and were subject to MitoSOX Red staining and flow cytometric analysis. (a) Representative histograms of cell counts (*counts*) vs. MitoSOX fluorescence (*FL2-H*) for un- (*left panel*) and infected cells (*right panel*) are shown. (b) The mean fluorescence intensity (MFI) of MitoSOX of mock- (−) and infected (+) cells is expressed as the percentage of that of uninfected cells. The results are presented as mean±SD, n = 3. *p<0.05 vs. uninfected cells.

Infection of neural SF268 cells with EV71 induces oxidative stress in time-dependent manner. The levels of ROS, as indicated by DCF fluorescence, in cells infected at multiplicity of infection (m.o.i.) of 2 were 3- and 6-fold that of uninfected control at 48 and 72 hr respectively (Figure 1). As mitochondria are major sites of ROS generation, we test if EV71 induces ROS generation in mitochondria. The EV71 infected-SF268 cells were stained at 54 hr post-infection (p.i.) with Mitotracker Red and H_2_DCFDA, and examined under confocal microscope. As expected, the DCF fluorescence increased in EV71-infected cell but not in the uninfected cells (Figures 2A & B). More important, the sites of ROS production coincided with mitochondria in EV71-infected cells (Figure 2B). It was consistent with staining of the infected cells with MitoSOX Red, a mitochondrion-specific probe for ROS (Figure 2C). The infected cells showed 3-fold increase in mitochondrial ROS generation. At infection time greater than 60 hr, the infected cells exhibited a great loss of viability. To study the host cell responses to viral infection, we focused on analyses of cells at early or mid-phase of infection in subsequent studies.

**Figure 3:**
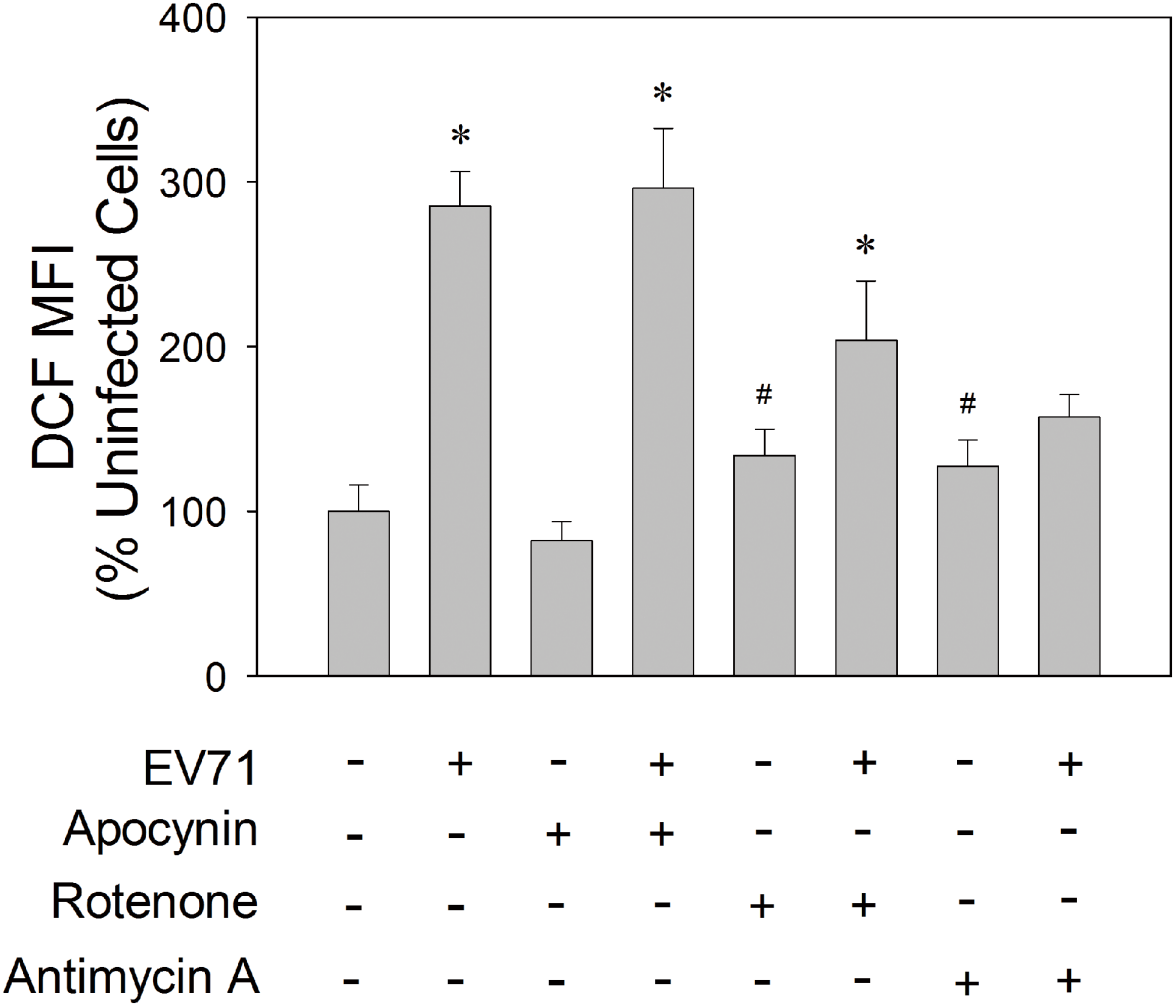
Mitochondrial electron transport-dependent ROS generation. SF268 cells were mock- or infected with EV71 at an m.o.i. of 1.25, and treated without or with apocyanin, rotenone or antimycin A. Forty-eight hours later, cells were stained with H_2_DCFDA and analyzed by flow cytometry. The MFI of DCF of infected cells is expressed as the percentage of that of uninfected cells. The results are presented as mean±SD, n = 3. *p<0.05 vs. untreated, uninfected cells; ^#^p<0.05, drug-treated vs. untreated cells.

The mitochondrial production of ROS is validated by pharmacological approach. Treatment of EV71-infected cells with complex I inhibitor rotenone and complex III inhibitor antimycin A significantly inhibited ROS generation (Figure 3). Unlike respiratory complex inhibitors, treatment of cells with apocyanin, a NADPH oxidase (NOX) inhibitor, did not affect EV71-induced ROS generation. These findings suggest that NOX is not involved in ROS generation in infected SF268 cells, and that ROS is generated at a site of mitochondrial electron transport chain distal to complex III.

### ROS production is associated with mitochondrial morphological anomalies

**Figure 4:**
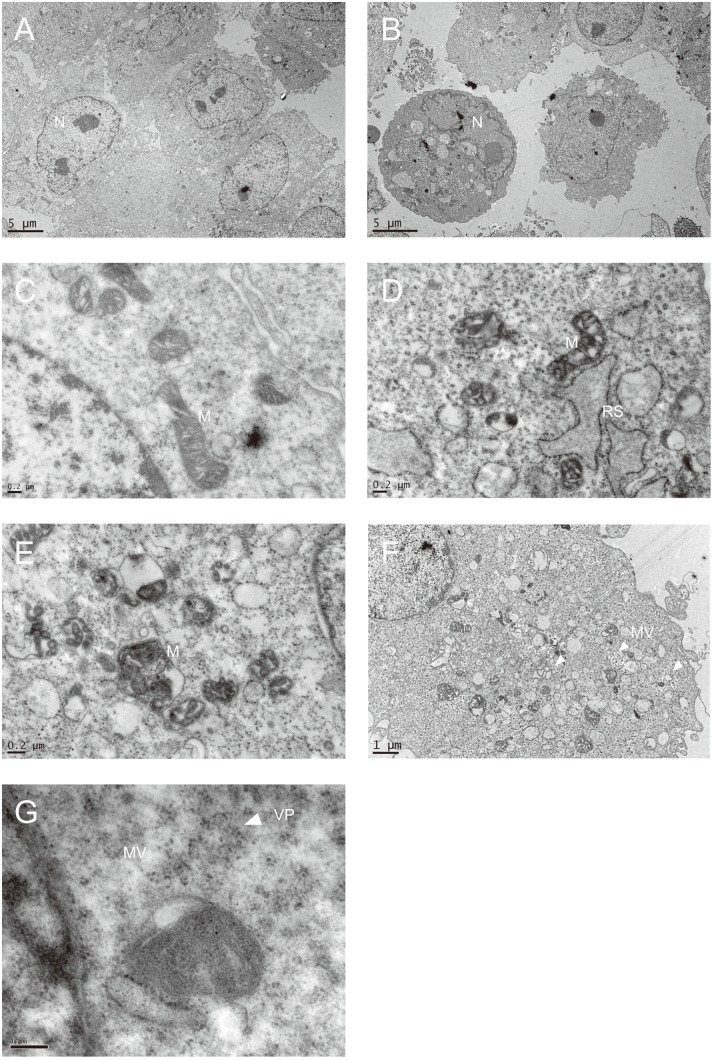
EV71 infection results in morphological changes in mitochondria. SF268 cells were mock- (A & C) or infected with (B, D–G) EV71 at an m.o.i. of 1.25 for 48 hr, and processed for electron microscopic examination. The mock-infected cells had typical nucleus (N) and mitochondria (M). In EV71-infected cells, a number of mitochondria underwent changes in morphology, characterized by deranged cristae (D & E). The developing viral replication site (RS) was lined with ribosomes and was in proximity to mitochondria (D). Numerous single or double membrane-bound vesicles (MV) developed in EV71-infected cells, and some contained virus particles (VP). For A & B, bar represents 5 µm; for F, bar represents 1 µm; for C, D, E & G, bar represents 0.2 µm.

Increased ROS production is suggestive of mitochondrial anomalies. To test such possibility, we examined the subcellular structure of infected SF268 cells using electron microscope. Forty-eight hour after infection, numerous SF268 cells were rounded. A number of membrane bound vesicle appeared in cytoplasm (Figures 4B & F). In some cells, replication sites of EV71 developed from rough endoplasmic reticulum, and were characterized by the presence of ribosomes at its periphery and accumulation of mitochondria in its proximity (Figure 4D). Smooth membrane bound vesicles were also observed. The replication sites and the virus-induced membrane vesicles were absent from the uninfected cells. A significant percentage of atypical mitochondria were observed. They were more electron-dense in infected cells as compared with control cells (Figure 4C & D). The crista arrangement in mitochondria of the infected cells became largely distorted (Figure 4D). The cristae were swollen and appeared as “holes” in electron micrograph. In some mitochondria, the crista structure was lost. The outer membrane was no longer closely apposed to the inner membrane, and the intermembrane space was enlarged (Figure 4E). Inner membrane was less conspicuous and even discontinuous in some mitochondria. These findings suggest that EV71 induces morphological anomalies in mitochondria.

### EV71 causes decline in mitochondrial electrochemical potential ΔΨ_m_

**Figure 5:**
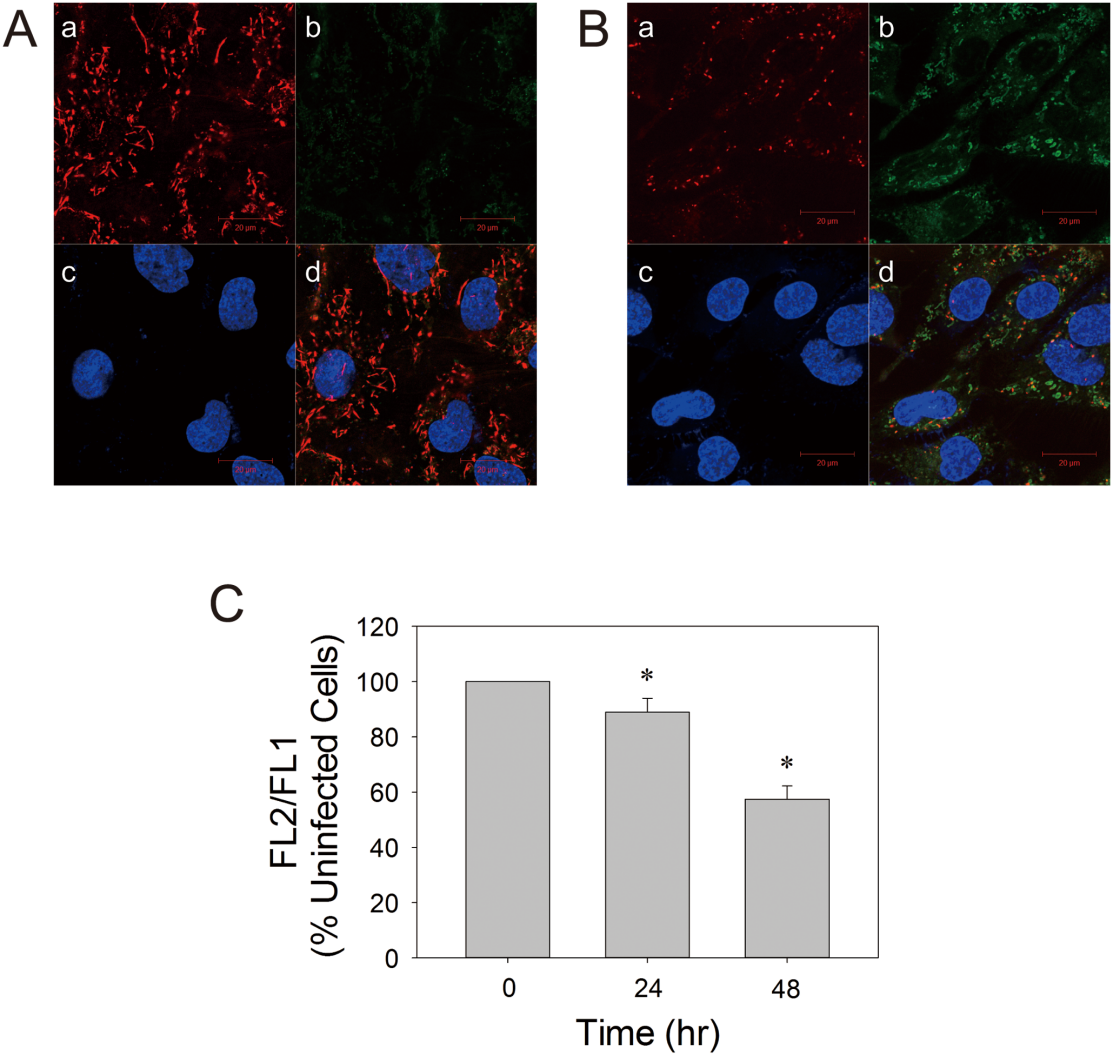
EV71 infection causes decline in ΔΨ_m_. SF268 cells were mock- (A) or infected with (B) EV71 at an m.o.i. of 1.25 for 48 hr. Cell were stained with JC-1 and Hoechst 33342 dyes, and examined by confocal microscopy. Intracellular distribution of JC-1 J-aggregate (a) and monomer (b) is indicative of ΔΨ_m_ in cells. Nuclei of these cells are shown (c). The corresponding images are overlaid (d). The photographs shown here are representative of three experiments. Scale bar, 20 µm. (C) SF268 cells were mock- or infected with EV71 at an m.o.i. of 1.25 for indicated times, and were subject to JC-1 staining and flow cytometric analysis. The ratio of MRI of FL2 channel to that of FL1 channel (FL2/FL1) was calculated, and is expressed relative to that of uninfected cells. Results are mean ± SD, n = 3. *p<0.05 vs. uninfected cells.

It is plausible that EV71 induces mitochondrial dysfunction in EV71-infected cells. A functional parameter of mitochondria is electrochemical potential ΔΨ_m_. We studied ΔΨ_m_ in infected cells using JC-1 staining. Mitochondria are heterogeneous in morphology, ranging from round, ovoid structure to long, interconnected structure. In uninfected cells, most mitochondria appeared red and represent energized mitochondria (Figure 5A). A large percentage of the energized mitochondria were elongated tubular or oblong structure. They formed a network distributed more evenly throughout cytoplasm. Upon infection, there were changes in mitochondria. Most mitochondria showed green fluorescence, and were depolarized or de-energized (Figure 5B). They appeared to cluster in the perinuclear region (Figures 2B & 5B). Some of mitochondria were swollen, and a majority of the remainder took a short ovoid form. Additionally, we applied a flow cytometric analysis to quantify ΔΨ_m_ in infected cells. Infection of cells with EV71 causes about 10 and 40% reduction in ΔΨ_m_ at 24 and 48 hr p.i., respectively. These findings suggest that EV71 infection induces significant drop in electrochemical potential.

### EV71-induced ROS generation and reduction in ΔΨ_m_ are associated with defective electron transport

**Figure 6:**
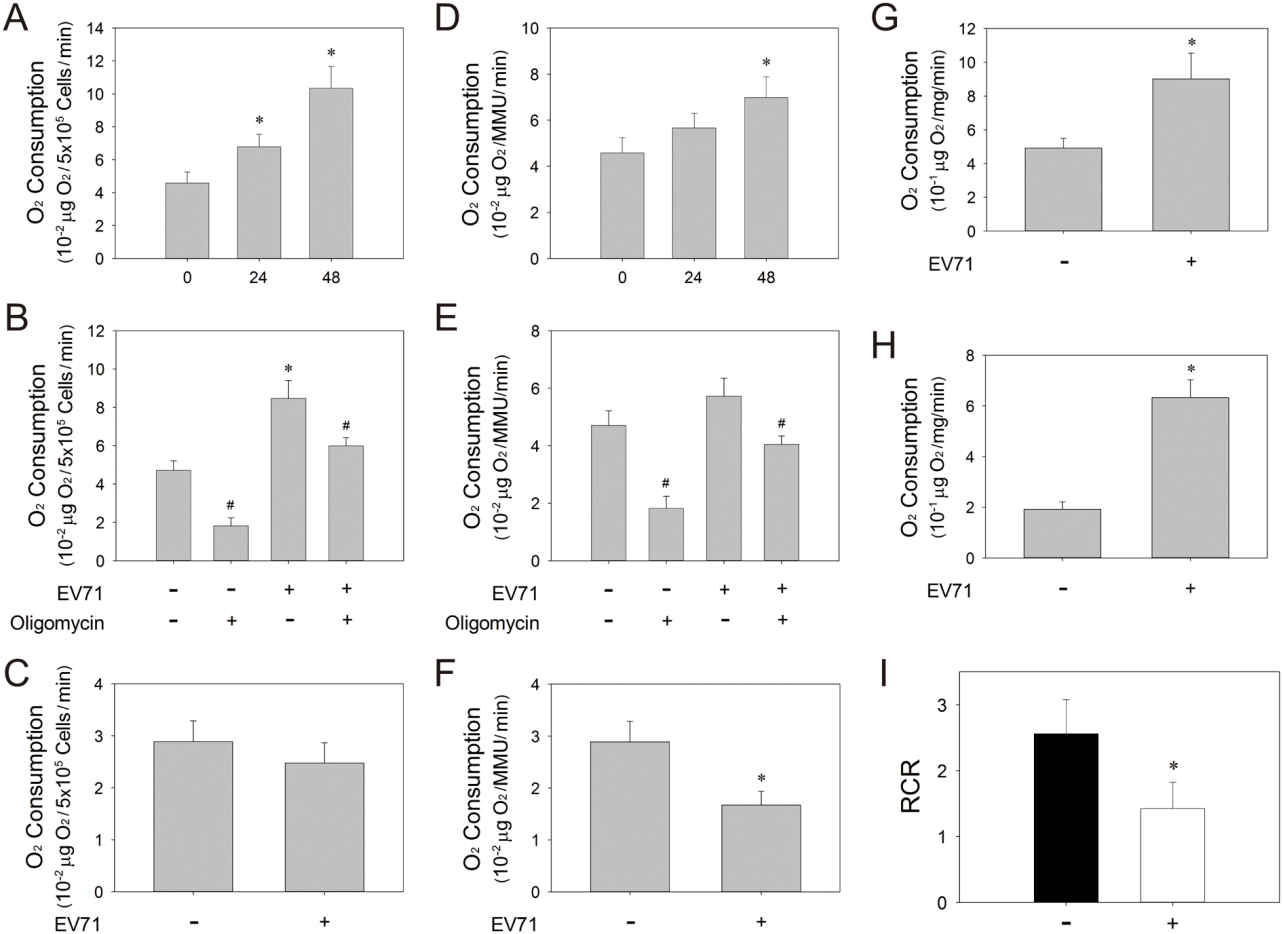
EV71 infection-induced oxygen consumption is associated with a reduction in respiratory efficiency. (A) SF268 cells were mock- or infected with EV71 at an m.o.i. of 1.25 for indicated times. Oxygen concentration was assayed with Clark oxygen electrode, and oxygen consumption rate (10^−2^ µg O_2_/5×10^5^ cells/min) was calculated accordingly. Results are mean ± SD, n = 6. *p<0.05 vs. uninfected cells. (B) SF268 cells were mock- or infected with EV71 at an m.o.i. of 1.25, and were treated without or with oligomycin. Oxygen concentration was assayed with Clark oxygen electrode, and oxygen consumption rate (10^−2^ µg O_2_/5×10^5^ cells/min) was calculated. Results are mean ± SD, n = 6. *p<0.05 vs. uninfected cells; ^#^p<0.05, oligomycin-treated vs. untreated cells. (C) Oligomycin-sensitive oxygen consumption rate was calculated as the difference in the absence and presence of oligomycin. (D & E) The oxygen consumption was measured as described in (A) and (B). Data were normalized to the relative mitochondrial mass unit (MMU) of control and infected cells. Results are mean ± SD, n = 6. *p<0.05 vs. uninfected cells. (F) Oligomycin-sensitive oxygen consumption rate was calculated as described in (C), and normalized to the relative mitochondrial mass unit (MMU) of control and infected cells. (G–I) SF268 cells were mock- (−) or infected (+) with EV71 at an m.o.i. of 1.25 for 48 hr, and mitochondria were isolated and assayed for oxygen consumption rates (10^−1^ µg O_2_/mg mitochondrial protein/min) during state 3 (G) and 4_o_ (H) respiration as described in *Materials and Methods*. RCRs were calculated accordingly, and are shown (I). Results are mean ± SD, n = 6. *p<0.05 vs. uninfected cells.

It is likely that increase in ROS generation and reduction in ΔΨ_m_ are causally related to anomalous electron transport. Amperometric method was applied to measure the oxygen consumption rate, which reflects the function of electron transport chain. As shown in Figure 6A, the oxygen consumption rate of SF268 cells increased after EV71 infection. The rate (normalized to cell number) was elevated about 2.3-fold at 48 hr p.i. However, the oligomycin-insensitive oxygen consumption (i.e. oxygen consumption in the presence of oligomycin), which represents that unrelated to ATP synthesis, accounted for a large percentage of oxygen consumption in infected cells. Oligomycin-insensitive oxygen consumption rate was about 38% of total oxygen consumption in control cells, while it increased about 3.3-fold and was up to 70% of total oxygen consumption in infected cells (Figure 6B). Oligomycin-sensitive oxygen consumption rates (i.e. difference in rates of oxygen consumption measured in the absence and presence of oligomycin, which represents oxygen used for oxidative phosphorylation) of control and infected cells were not significantly different (Figure 6C). A different picture emerged when the oxygen consumption rates were normalized to the relative mitochondrial mass of control and infected cells (Figure 6D–F). The oligomycin-sensitive oxygen consumption rate of infected cells was 42% lower than that of control cells (Figure 6F). Furthermore, we measured state 3 and 4_o_ respiration of isolated mitochondria from control and EV71-infected cells (Figure 6G–I), and calculated the respective respiratory control ratio (RCR), which is considered an index of electron transport coupling. As shown in Figure 6I, it was 44% lower for mitochondria from infected cells than for those from control cells.

### EV71 infection causes an increase in mitochondrial mass and alteration in protein expression

**Figure 7:**
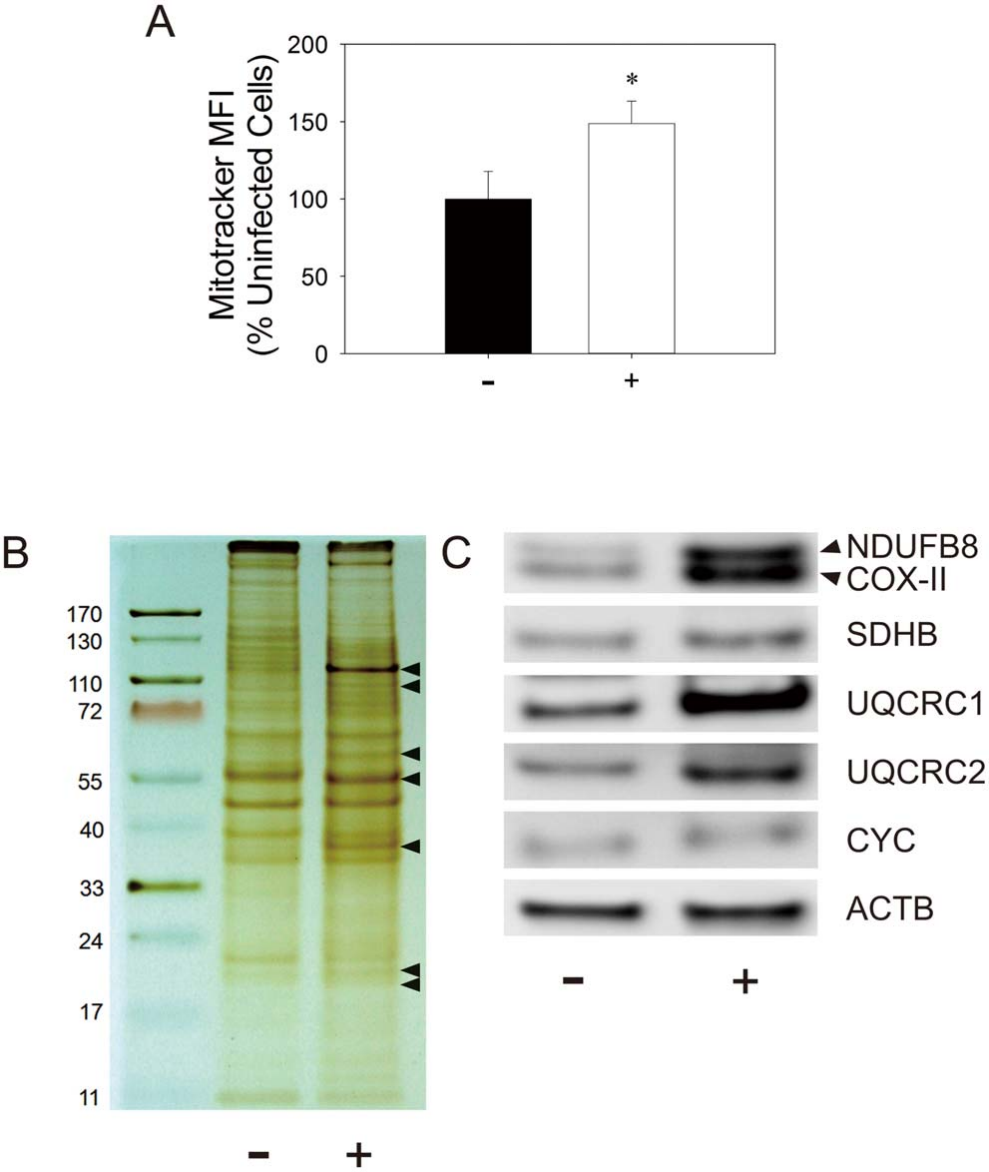
Mitochondrial mass increases and expression of mitochondrial proteins changes in response to EV71 infection. (A) SF268 cells were mock- (−) or infected (+) with EV71 at an m.o.i. of 1.25 for 48 hr, and were subject to Mitotracker dye staining and flow cytometric analysis as described in *Materials and Methods*. The MFI of the stained cells is expressed relative to that of control cells. Results are mean ± SD, n = 3. *p<0.05 vs. uninfected cells. (B & C) Cells were un- or infected under the similar condition, and mitochondria were isolated for SDS-PAGE electrophoresis and silver staining (B). In the silver-stained gel, the leftmost lane corresponds to protein markers with respective molecular weights indicated alongside the bands. (C) Cells similarly infected were harvested for western blotting with indicated antibodies. A representative experiment out of three is shown here.

There appears to be a discrepancy between oxygen consumption rates normalized to cell number and mitochondrial mass. It is possible that EV71-infected cells have a compensatory increase in mitochondrial mass. To test such possibility, we stained control and infected cells with mitochondrion-specific dye, and analyzed them cytometrically for quantification of mitochondrial mass. As shown in Figure 7A, mitochondrial mass of EV71-infected cells at 48 hr p.i. was about 50% higher than that of control. It was accompanied by an increased expression of mitochondrial proteins. Figure 7B shows the silver-stained SDS-PAGE of mitochondrial preparations from control and infected cells. Intensities of a number of bands increased in infected cells, which is probably indicative of enhanced expression of mitochondrial proteins. Additionally, some proteins were differentially expressed in control and infected cells. Furthermore, we determined the expression of specific mitochondrial proteins using immunoblotting. Expression of such mitochondrial proteins as NADH dehydrogenase (ubiquinone) 1 β subcomplex 8 (NDUFB8), ubiquinol-cytochrome C reductase core protein I (UQCRC1), ubiquinol-cytochrome C reductase core protein II (UQCRC2), and cytochrome c oxidase II (COX-II) was elevated. Other proteins, for example succinate dehydrogenase complex subunit B iron sulfur protein (SDHB) and cytochrome C (CYC), remained largely unchanged in their expression. These findings suggest that EV71 infection induces mitochondrial proliferation and changes in expression of mitochondrial proteins.

### Mitochondrial dysfunction is associated with anomalous metabolism of adenine nucleotides

**Figure 8:**
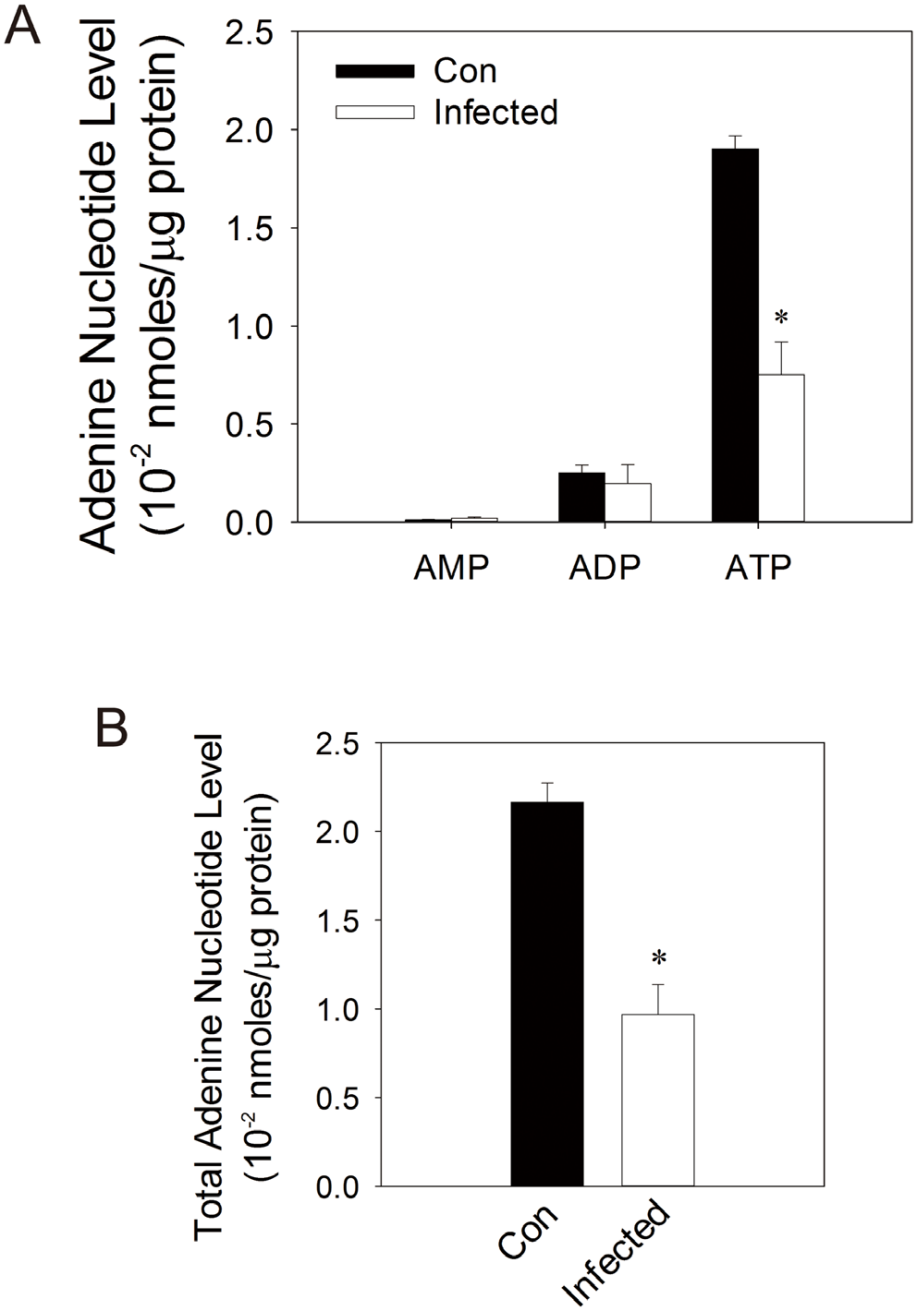
Levels of ATP, ADP and AMP in EV71-infected cells. SF268 cells were mock- (*Con*) or infected (*Infected*) with EV71 at an m.o.i. of 1.25 for 48 hr, and were harvested for UPLC-based analyses of ATP, ADP and AMP. These adenine nucleotides are normalized to cellular protein content. Levels of ATP, ADP and AMP (A) and total adenine nucleotides (B) are shown. Results are mean ± SD, n = 3. *p<0.05 vs. uninfected cells.

It is possible that mitochondrial dysfunction affects ATP supply. We measured the levels of ATP, ADP and AMP in control and infected cells. As shown in Figure 8, intracellular level of ATP was reduced by about 60% in infected cells, while those of ADP and AMP remained largely unchanged. Total adenine nucleotides decreased in infected cells. The energy charge (
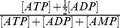
) of control and infected cells were 0.94 and 0.87, respectively. These findings suggest that EV71 infection results in lower energy status of infected cells.

### Mitochondrial ROS generation is essential to EV71 replication

**Figure 9:**
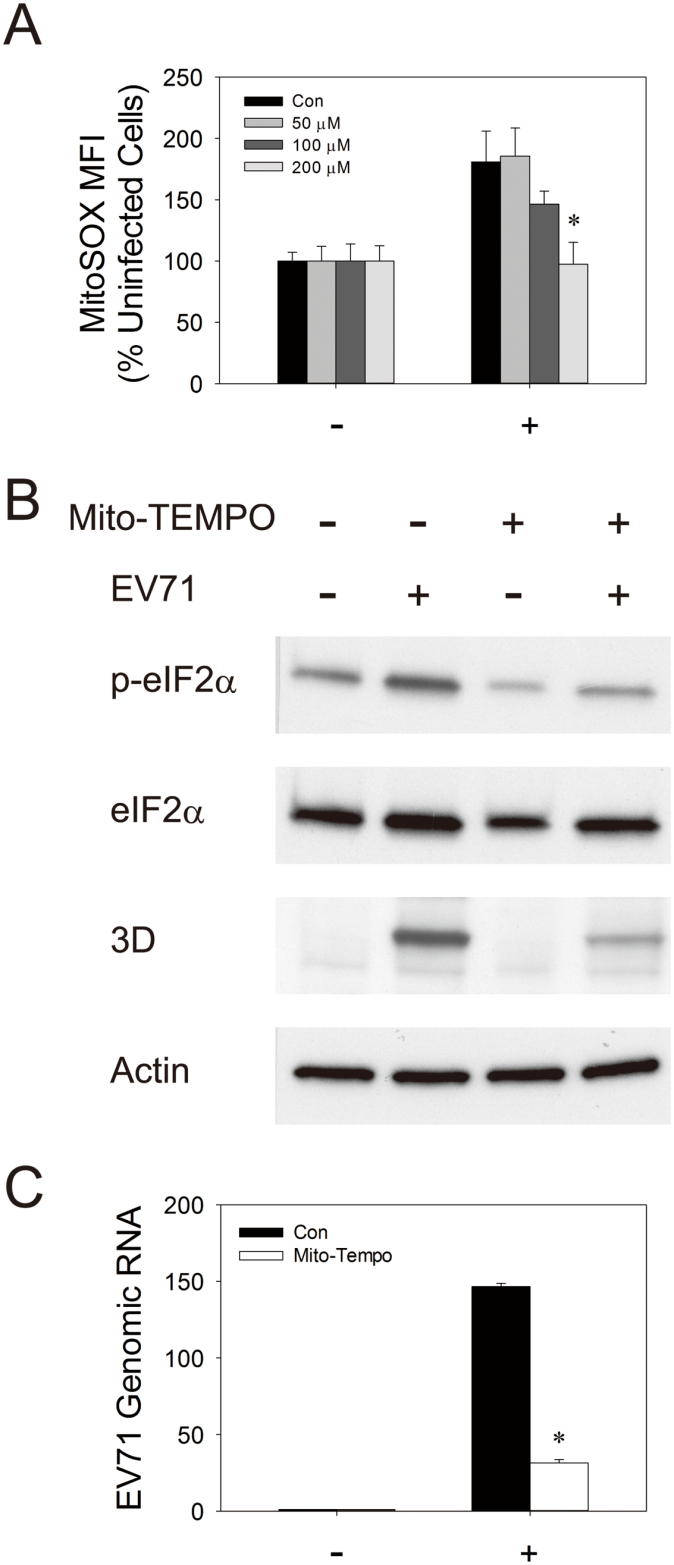
Mitochondrial ROS are essential to EV71 replication. (A) SF268 cells were mock- (−) or infected (+) with EV71 at an m.o.i. of 1.25, and treated without (*Con*) or with indicated concentrations of Mito-TEMPO. Forty-eight hours later, cells were subject to MitoSOX Red staining and flow cytometric analysis. The mean fluorescence intensity (MFI) of MitoSOX of mock- and infected cells is expressed as the percentage of that of uninfected cells. The results are presented as mean ± SD, n = 3. *p<0.05 vs. infected Con group. (B) SF268 cells were mock- or infected with EV71 at an m.o.i. of 1.25, and treated without or with 200 µM of Mito-TEMPO. Forty-eight hours later, cells were harvested for western blotting with antibodies to phosphorylated eIF2α and total eIF2α, viral protein 3D, and actin. A representative experiment out of three is shown here. (C) SF268 cells were mock- (−) or infected (+) with EV71 at an m.o.i. of 1.25, and treated without (*Con*) or with 200 µM of Mito-TEMPO. Forty-eight hours later, cells were analyzed for levels of EV71 genomic RNA. The results are presented as means ± SD n = 3. *p<0.05 vs. infected Con group.

We have previously shown that ROS are conducive to viral replication. It is possible that mitochondrion-generated ROS act as signal to initiate molecular events associated with EV71 replication. We treated control and uninfected cells with mitochondrion-specific antioxidant mito-TEMPO, and studied its consequences on EV71 infection. To determine the optimal mito-TEMPO concentration for experiments, we treated control and EV71-infected cells with increasing concentrations of mito-TEMPO, and studied its effect on ROS generation. Mito-TEMPO reduced EV71-induced mitochondrial ROS generation in a dose-dependent manner (Figure 9A). At 200 µM, mito-TEMPO completely suppressed mitochondrial ROS production in infected cells. This concentration was used in subsequent experiments. It is known that phosphorylation of eIF2α signifies the initiation of viral protein synthesis during enteroviral infection [44], [45]. We treated control and EV71-infected cells with 200 µM mito-TEMPO, and examined the effect of mito-TEMPO on eIF2α phosphorylation. As shown in Figure 9B, the level of eIF2α phosphorylation increased significantly in infected cells. Treatment with mito-TEMPO reduced both basal and EV71-induced eIF2α phosphorylation. It was associated with significant reduction in expression of viral 3D protein and viral replication (Figures 9B & C). At 48 hr p.i., the copy number of viral genome in cells treated with mito-TEMPO was about 80% lower than that of untreated cells (Figure 9C). These findings suggest that mitochondrial ROS are essential to EV71 replication.

## Discussion

In the present study, we demonstrate that EV71 induces mitochondrial dysfunction and ROS generation, which acts to promote replication. There appears to be increases in mitochondrial mass, which probably represents a mechanism to compensate for mitochondrial defects.

EV71 induces generation of ROS, which in turn promotes viral replication [16]. The co-localization of fluorescence signals of Mitotracker Red and DCF as well as the increase in fluorescence of MitoSox Red in infected cells suggest that ROS generated in EV71-infected cells are of mitochondrial origin. Previous study has shown that EV71 activates Rac1-dependent NADPH oxidase [46]. The findings that treatment of infected cells with rotenone and antimycin A but not with apocyanin inhibited ROS generation are suggestive of ROS generation through inadvertent reaction between electron passing down electron transport chain and oxygen. Moreover, ROS appear to be generated at a point of electron transport chain downstream of complex III. For instance, cytochrome C can transfer electron directly to p66^Shc^, a redox enzyme, to generate ROS [47]. The heme center of cytochrome C may also act to enhance ROS production [48].

The mechanism for EV71-induced ROS production is currently enigmatic. A number of viral proteins interact with mitochondria or their proteins, and causes mitochondrial dysfunction and ROS generation. For example, hepatitis B virus X protein (HBx) interacts with mitochondrial heat shock protein 60 and 70, and VDAC3 [29], [49], and promotes oxidative stress [50]. HCV core protein binds to mitochondria and increases oxidative stress [51]–[53]. Influenza A virus PB1-F2 protein targets to mitochondria and induces their anomalies [54], [55]. Our preliminary results have recently shown that EV71 proteins associate with mitochondria and elicit oxidative stress.

The functional significance of ROS in EV71 remains unclear. It is undisputable that ROS generation has functional consequence on viral infection. Rabies virus can induce oxidative stress in dorsal root ganglion neurons, which diminishes axonal growth [56]. Oxidative stress also promotes S-glutathionylation of cellular proteins, which regulates activities of various cellular proteins [57]. It has been shown that de-glutathionylation of interferon regulatory factor 3 is required for its efficient interaction with CBP and transactivation of interferon genes [58]. Treatment with mito-TEMPO that reduces mitochondrial ROS generation inhibits eIF2α phosphorylation, a hallmark event in EV71 infection. It is probable that ROS play a regulatory role in EV71 infection.

Mitochondria play a prominent role in energy metabolism. Electron transport through respiratory complexes and the resulting proton motive force are critical to oxidative phosphorylation. Virus-induced mitochondrial dysfunction is not specific for EV71 and can be observed for other viruses. In most cases, viral infection induces mitochondrial dysfunction, and reduction in proton motive force and cellular ATP content. Hepatitis C virus (HCV)-infected cells show mitochondrial dysfunction and decrease in ΔΨ_m_ [59]. Infection of mouse neural cells with Sindbis virus causes mitochondrial dysfunction [60]. The lowered mitochondrial respiratory efficiency of EV71-infected cells is consistent with change in cristae. It has been recently shown that cristae shape affects the interaction between respiratory complexes and the respiratory efficiency [61]. Decrease in RCR is accompanied by decrease in ΔΨ_m_. A drop in ΔΨ_m_ causes a deficit in oxidative phosphorylation, which is indicated by decreases in energy charge and ATP/ADP ratio. Moreover, it has been recently shown that dissipation of proton motive force leads to defective MAVS signaling and hence crippled antiviral defense [62], [63].

The subcellular distribution of mitochondria in EV71-infected cells changes after infection. After infection, the mitochondria are converted from the interconnecting tubular form to short ovoid form, and are clustered near nuclei. Perinuclear clustering of mitochondria has also been observed for HBV and HCV [64], [65]. It is reasoned that re-distribution of mitochondria in infected cells serves the purpose either to meet the energy requirement of viral infection process or to cordon off mitochondria to prevent pro-apoptotic mediators.

Energy is needed for synthesis of viral protein and nucleic acid. Changes in metabolism in host cells have been implicated in viral pathogenesis [66]. The depletion of ATP during EV71 infection is attributed to its direct incorporation into newly synthesized viral genome, or to its involvement in energy-requiring processes. Decrease in RCR has much impact on cellular metabolism. To ensure the continual ATP supply to support viral replication process, the infected cells may put a strain on “shop-worn” but still working mitochondria for energy production, and may activate glycolytic pathway to meet such need. Our preliminary results have recently shown that glycolytic pathway may be enhanced in EV71-infected cells. The decrease in respiratory efficiency of mitochondria, together with the low ATP-generating efficiency of glycolysis, results in reduction in energy charge. Moreover, mitochondria are involved in biosynthesis of such biomolecules as lipids. Picornaviral replication machinery is assembled on membrane, which is probably derived from endoplasmic reticulum [67]. Such process depends on significant alteration of membrane lipids. Alteration of mitochondria during infection may shift the lipid metabolic reactions, such as phosphatidylserine decarboxylation, cytochrome P450-dependent steroid metabolism or acetyl-CoA/citrate metabolism, to fulfill the metabolic need associated with viral replication. It has been recently shown that poliovirus induces long chain acyl-CoA synthetase 3 activity [68], the mitochondrial outer membrane protein that regulates fatty acid import for formation of replication complex. It is envisaged that impaired mitochondria are functionally altered to sustain viral replication.

As mentioned in preceding paragraphs, functional alterations of mitochondria may play vital roles during EV71 infection. Mitochondria undergo adaptive changes to compensate for their reduced respiratory efficiency and diminished functions. A non-exclusive view is that different pools of mitochondria exist [69], [70]. Some mitochondria may experience different extent of dysfunctional change. The damaged but still-functional mitochondria may undergo biogenesis and make up for the deficit in mitochondrial functions. The increases in mitochondrial mass may represent such adaptive response. It is not unprecedented that virus induces mitochondrial biogenesis. For instance, human cytomegalovirus infection causes biogenesis of mitochondria [71]. Accumulation of mitochondria in the vicinity of viral assembly factories in African swine fever virus-infected cells is accompanied by *de novo* synthesis of mitochondria mass [72]. Increased mitochondrial mass in EV71-infected cells is accompanied by differential increases in levels of mitochondrial proteins. Mitochondrial proteins, such as COX-II, are specifically elevated. Increase in level of COX-II is consistent with increase in its transcription [73]. Interestingly, levels of mitochondrial proteins are differentially changed during infection. It is speculative whether such differential changes in protein expression contribute to mitochondrial respiratory functions. It has been recently suggested that the respiratory chain complexes are not arranged simply in a linear order of enzymatic complexes. These complexes display different activities toward one another [74]. Subunits of respiratory complexes differ in their correlation between protein expression and enzymatic activity [75]. It is exemplified by the 8 kDa subunit of complex I, for which one-thirds of protein level correlates with 70% of complex I activity. It follows that differential increases in mitochondrial proteins can contribute to maintenance of respiratory functions.

Previous studies have shown that EV71 infection elicits apoptosis in SF268 cells [76]. Conspicuous apoptosis is detected at 72 hr post-infection. However, apoptosis is atypical in the sense that Bid is not cleaved in EV71-infected SF268 cells. tBid, the cleavage product of Bid, is involved in permeabilization of mitochondrial outer membrane [77]. It is currently unknown whether mitochondrial membrane permeabilization is involved in EV71-induced death of SF268 cells. It is likely that mitochondria in these cells differ in some ways from those of infected non-neural cells, which exhibit typical apoptosis. Moreover, our studies focus on the early or mid-phase of infection. It is conceivable that mitochondria can undergo biogenesis in SF268 cells at early or mid-phase of infection, whilst they experience destructive changes during the late phase of infection.

Vesicular structures in picornavirus-infected cells represent the site of replication. These membranous structures are thought to be derived from endoplasmic reticulum (ER). Synthesis of picornaviral protein can activate ER stress response. It has been recently shown that EV71 induces ER stress response and causes eIF2α phosphorylation through activation of PKR and PERK [44]. Phosphorylation of eIF2α suppresses cap-dependent translation, causing a shift in translation to that of internal ribosome-entry site (IRES)-containing cellular mRNA and enteroviral RNA [78]. Interestingly, ROS are implicated in activation of PKR and PERK [79]–[83], raising the possibility that mitochondrial ROS may play signaling role in the process.

Antioxidants may be used as therapeutic intervention of enteroviral infection. We have previously shown that *N*-acetylcysteine and epigallocatechin gallate can suppress EV71 replication [16], [35]. The antiviral activities of epigallocatechin gallate and related compounds correlate with their antioxidative activities. The fact that mito-TEMPO has antiviral effect pinpoints mitochondrial ROS as target for therapeutic intervention.

Taken together, EV71 causes alterations in mitochondria to induce ROS generation. The mitochondrial ROS are essential to viral replication. The “dysfunction” of mitochondria, such as decline in respiratory functions, may represent virus-induced functional alterations, which are optimized for viral replication. Mitochondria may undergo adaptive or compensatory increase in mitochondrial mass to serve such purpose.

## References

[1] Racaniello VR (2001) Picornaviridae: the viruses and their replication. In: Fields BN, Knipe DM, Howley PM, Griffin DE, editors. Fields’ Virology. Philadelphia: Lippincott Williams & Wilkins. 685–722.

[2] Schmidt NJ, Lennette EH, Ho HH (1974) An apparently new enterovirus isolated from patients with disease of the central nervous system. J Infect Dis 129: 304–309. 4361245 10.1093/infdis/129.3.304

[3] Ho M, Chen ER, Hsu KH, Twu SJ, Chen KT, et al (1999) An epidemic of enterovirus 71 infection in Taiwan. Taiwan Enterovirus Epidemic Working Group. N Engl J Med 341: 929–935. 10498487 10.1056/NEJM199909233411301

[4] Yang F, Ren L, Xiong Z, Li J, Xiao Y, et al (2009) Enterovirus 71 outbreak in the People’s Republic of China in 2008. J Clin Microbiol 47: 2351–2352. 19439545 10.1128/JCM.00563-09 PMC2708525

[5] Yang F, Zhang T, Hu Y, Wang X, Du J, et al (2011) Survey of enterovirus infections from hand, foot and mouth disease outbreak in China, 2009. Virol J 8: 508. 22054534 10.1186/1743-422X-8-508 PMC3227625

[6] McMinn PC (2002) An overview of the evolution of enterovirus 71 and its clinical and public health significance. FEMS Microbiol Rev 26: 91–107. 12007645 10.1111/j.1574-6976.2002.tb00601.x

[7] Ishimaru Y, Nakano S, Yamaoka K, Takami S (1980) Outbreaks of hand, foot, and mouth disease by enterovirus 71. High incidence of complication disorders of central nervous system. Arch Dis Child 55: 583–588. 6254449 10.1136/adc.55.8.583 PMC1627055

[8] Lin TY, Chang LY, Hsia SH, Huang YC, Chiu CH, et al (2002) The 1998 enterovirus 71 outbreak in Taiwan: pathogenesis and management. Clin Infect Dis 34 Suppl 2: S52–57. 11938497 10.1086/338819

[9] Ooi MH, Wong SC, Lewthwaite P, Cardosa MJ, Solomon T (2010) Clinical features, diagnosis, and management of enterovirus 71. Lancet Neurol 9: 1097–1105. 20965438 10.1016/S1474-4422(10)70209-X

[10] Huang CC, Liu CC, Chang YC, Chen CY, Wang ST, et al (1999) Neurologic complications in children with enterovirus 71 infection. N Engl J Med 341: 936–942. 10498488 10.1056/NEJM199909233411302

[11] Beck MA, Handy J, Levander OA (2000) The role of oxidative stress in viral infections. Ann N Y Acad Sci 917: 906–912. 11268420 10.1111/j.1749-6632.2000.tb05456.x

[12] Beck MA, Levander OA, Handy J (2003) Selenium deficiency and viral infection. J Nutr 133: 1463S–1467S. 12730444 10.1093/jn/133.5.1463S

[13] Cai J, Chen Y, Seth S, Furukawa S, Compans RW, et al (2003) Inhibition of influenza infection by glutathione. Free Radic Biol Med 34: 928–936. 12654482 10.1016/s0891-5849(03)00023-6

[14] Beck MA, Shi Q, Morris VC, Levander OA (2005) Benign coxsackievirus damages heart muscle in iron-loaded vitamin E-deficient mice. Free Radic Biol Med 38: 112–116. 15589379 10.1016/j.freeradbiomed.2004.10.007

[15] Aquaro S, Muscoli C, Ranazzi A, Pollicita M, Granato T, et al (2007) The contribution of peroxynitrite generation in HIV replication in human primary macrophages. Retrovirology 4: 76. 17949509 10.1186/1742-4690-4-76 PMC2173904

[16] Ho HY, Cheng ML, Weng SF, Chang L, Yeh TT, et al (2008) Glucose-6-phosphate dehydrogenase deficiency enhances enterovirus 71 infection. J Gen Virol 89: 2080–2089. 18753216 10.1099/vir.0.2008/001404-0

[17] Oda T, Akaike T, Hamamoto T, Suzuki F, Hirano T, et al (1989) Oxygen radicals in influenza-induced pathogenesis and treatment with pyran polymer-conjugated SOD. Science 244: 974–976. 2543070 10.1126/science.2543070

[18] Schachtele SJ, Hu S, Little MR, Lokensgard JR (2010) Herpes simplex virus induces neural oxidative damage via microglial cell Toll-like receptor-2. J Neuroinflammation 7: 35. 20584314 10.1186/1742-2094-7-35 PMC2904293

[19] Kaul P, Biagioli MC, Singh I, Turner RB (2000) Rhinovirus-induced oxidative stress and interleukin-8 elaboration involves p47-phox but is independent of attachment to intercellular adhesion molecule-1 and viral replication. J Infect Dis 181: 1885–1890. 10837166 10.1086/315504 PMC7109975

[20] Mochizuki H, Todokoro M, Arakawa H (2009) RS virus-induced inflammation and the intracellular glutathione redox state in cultured human airway epithelial cells. Inflammation 32: 252–264. 19548075 10.1007/s10753-009-9128-0

[21] Tal MC, Iwasaki A (2011) Mitoxosome: a mitochondrial platform for cross-talk between cellular stress and antiviral signaling. Immunol Rev 243: 215–234. 21884179 10.1111/j.1600-065X.2011.01038.x PMC3170140

[22] Ohta A, Nishiyama Y (2011) Mitochondria and viruses. Mitochondrion 11: 1–12. 20813204 10.1016/j.mito.2010.08.006 PMC7105242

[23] Kuwana T, Newmeyer DD (2003) Bcl-2-family proteins and the role of mitochondria in apoptosis. Curr Opin Cell Biol 15: 691–699. 14644193 10.1016/j.ceb.2003.10.004

[24] Brunelle JK, Letai A (2009) Control of mitochondrial apoptosis by the Bcl-2 family. J Cell Sci 122: 437–441. 19193868 10.1242/jcs.031682 PMC2714431

[25] Piccoli C, Quarato G, Ripoli M, D’Aprile A, Scrima R, et al (2009) HCV infection induces mitochondrial bioenergetic unbalance: causes and effects. Biochim Biophys Acta 1787: 539–546. 19094961 10.1016/j.bbabio.2008.11.008

[26] Wang T, Weinman SA (2006) Causes and consequences of mitochondrial reactive oxygen species generation in hepatitis C. J Gastroenterol Hepatol. 21 Suppl 3: S34–37. 10.1111/j.1440-1746.2006.04591.x 16958669

[27] Piccoli C, Scrima R, Quarato G, D’Aprile A, Ripoli M, et al (2007) Hepatitis C virus protein expression causes calcium-mediated mitochondrial bioenergetic dysfunction and nitro-oxidative stress. Hepatology 46: 58–65. 17567832 10.1002/hep.21679

[28] Lecoeur H, Borgne-Sanchez A, Chaloin O, El-Khoury R, Brabant M, et al (2012) HIV-1 Tat protein directly induces mitochondrial membrane permeabilization and inactivates cytochrome c oxidase. Cell Death Dis 3: e282. 22419111 10.1038/cddis.2012.21 PMC3317353

[29] Rahmani Z, Huh KW, Lasher R, Siddiqui A (2000) Hepatitis B virus X protein colocalizes to mitochondria with a human voltage-dependent anion channel, HVDAC3, and alters its transmembrane potential. J Virol 74: 2840–2846. 10684300 10.1128/jvi.74.6.2840-2846.2000 PMC111774

[30] Li Q, Wang L, Dong C, Che Y, Jiang L, et al (2005) The interaction of the SARS coronavirus non-structural protein 10 with the cellular oxido-reductase system causes an extensive cytopathic effect. J Clin Virol 34: 133–139. 16157265 10.1016/j.jcv.2004.12.019 PMC7108382

[31] Lachgar A, Sojic N, Arbault S, Bruce D, Sarasin A, et al (1999) Amplification of the inflammatory cellular redox state by human immunodeficiency virus type 1-immunosuppressive tat and gp160 proteins. J Virol 73: 1447–1452. 9882350 10.1128/jvi.73.2.1447-1452.1999 PMC103969

[32] Waris G, Huh KW, Siddiqui A (2001) Mitochondrially associated hepatitis B virus X protein constitutively activates transcription factors STAT-3 and NF-kappa B via oxidative stress. Mol Cell Biol 21: 7721–7730. 11604508 10.1128/MCB.21.22.7721-7730.2001 PMC99943

[33] Rutka JT, Giblin JR, Dougherty DY, Liu HC, McCulloch JR, et al (1987) Establishment and characterization of five cell lines derived from human malignant gliomas. Acta Neuropathol 75: 92–103. 2829496 10.1007/BF00686798

[34] Kornblith PL, Szypko PE (1978) Variations in response of human brain tumors to BCNU in vitro. J Neurosurg 48: 580–586. 632883 10.3171/jns.1978.48.4.0580

[35] Ho HY, Cheng ML, Weng SF, Leu YL, Chiu DT (2009) Antiviral effect of epigallocatechin gallate on enterovirus 71. J Agric Food Chem 57: 6140–6147. 19537794 10.1021/jf901128u

[36] Ho HY, Cheng ML, Chiu HY, Weng SF, Chiu DT (2008) Dehydroepiandrosterone induces growth arrest of hepatoma cells via alteration of mitochondrial gene expression and function. Int J Oncol 33: 969–977. 18949359

[37] Cheng ML, Shiao MS, Chiu DT, Weng SF, Tang HY, et al (2011) Biochemical disorders associated with antiproliferative effect of dehydroepiandrosterone in hepatoma cells as revealed by LC-based metabolomics. Biochem Pharmacol 82: 1549–1561. 21843511 10.1016/j.bcp.2011.07.104

[38] Moran M, Rivera H, Sanchez-Arago M, Blazquez A, Merinero B, et al (2010) Mitochondrial bioenergetics and dynamics interplay in complex I-deficient fibroblasts. Biochim Biophys Acta 1802: 443–453. 20153825 10.1016/j.bbadis.2010.02.001

[39] Kristian T, Hopkins IB, McKenna MC, Fiskum G (2006) Isolation of mitochondria with high respiratory control from primary cultures of neurons and astrocytes using nitrogen cavitation. J Neurosci Methods 152: 136–143. 16253339 10.1016/j.jneumeth.2005.08.018 PMC2572758

[40] Schrand AM, Schlager JJ, Dai L, Hussain SM (2010) Preparation of cells for assessing ultrastructural localization of nanoparticles with transmission electron microscopy. Nat Protoc 5: 744–757. 20360769 10.1038/nprot.2010.2

[41] Cheng ML, Ho HY, Lin HY, Lai YC, Chiu DT (2013) Effective NET formation in neutrophils from individuals with G6PD Taiwan-Hakka is associated with enhanced NADP(+) biosynthesis. Free Radic Res 47: 699–709. 23777333 10.3109/10715762.2013.816420

[42] Lin CJ, Ho HY, Cheng ML, You TH, Yu JS, et al (2010) Impaired dephosphorylation renders G6PD-knockdown HepG2 cells more susceptible to H(2)O(2)-induced apoptosis. Free Radic Biol Med 49: 361–373. 20420899 10.1016/j.freeradbiomed.2010.04.019

[43] Shevchenko A, Wilm M, Vorm O, Mann M (1996) Mass spectrometric sequencing of proteins silver-stained polyacrylamide gels. Anal Chem 68: 850–858. 8779443 10.1021/ac950914h

[44] Jheng JR, Lau KS, Tang WF, Wu MS, Horng JT (2010) Endoplasmic reticulum stress is induced and modulated by enterovirus 71. Cell Microbiol 12: 796–813. 20070307 10.1111/j.1462-5822.2010.01434.x

[45] Hanson PJ, Zhang HM, Hemid MG, Ye X, Qiu Y, et al.. (2013) Viral Replication Strategies: Manipulation of ER Stress Response Pathways and Promotion of IRES-Dependent Translation. In: Rosas-Acosta G, editor. Viral Replication. Croatia: Intech. 103–126.

[46] Tung WH, Hsieh HL, Lee IT, Yang CM (2011) Enterovirus 71 induces integrin beta1/EGFR-Rac1-dependent oxidative stress in SK-N-SH cells: role of HO-1/CO in viral replication. J Cell Physiol 226: 3316–3329. 21321939 10.1002/jcp.22677

[47] Giorgio M, Migliaccio E, Orsini F, Paolucci D, Moroni M, et al (2005) Electron transfer between cytochrome c and p66Shc generates reactive oxygen species that trigger mitochondrial apoptosis. Cell 122: 221–233. 16051147 10.1016/j.cell.2005.05.011

[48] Akopova OV, Kolchinskaya LI, Nosar VI, Bouryi VA, Mankovska IN, et al (2012) Cytochrome C as an amplifier of ROS release in mitochondria. Fiziol Zh 58: 3–12. 22586905

[49] Zhang SM, Sun DC, Lou S, Bo XC, Lu Z, et al (2005) HBx protein of hepatitis B virus (HBV) can form complex with mitochondrial HSP60 and HSP70. Arch Virol 150: 1579–1590. 15789261 10.1007/s00705-005-0521-1

[50] Anand SK, Tikoo SK (2013) Viruses as modulators of mitochondrial functions. Adv Virol 2013: 738794. 24260034 10.1155/2013/738794 PMC3821892

[51] Koike K (2007) Hepatitis C virus contributes to hepatocarcinogenesis by modulating metabolic and intracellular signaling pathways. J Gastroenterol Hepatol 22 Suppl 1: S108–111. 17567457 10.1111/j.1440-1746.2006.04669.x

[52] Paracha UZ, Fatima K, Alqahtani M, Chaudhary A, Abuzenadah A, et al (2013) Oxidative stress and hepatitis C virus. Virol J 10: 251. 23923986 10.1186/1743-422X-10-251 PMC3751576

[53] Schwer B, Ren S, Pietschmann T, Kartenbeck J, Kaehlcke K, et al (2004) Targeting of hepatitis C virus core protein to mitochondria through a novel C-terminal localization motif. J Virol 78: 7958–7968. 15254168 10.1128/JVI.78.15.7958-7968.2004 PMC446112

[54] Gibbs JS, Malide D, Hornung F, Bennink JR, Yewdell JW (2003) The influenza A virus PB1-F2 protein targets the inner mitochondrial membrane via a predicted basic amphipathic helix that disrupts mitochondrial function. J Virol 77: 7214–7224. 12805420 10.1128/JVI.77.13.7214-7224.2003 PMC164823

[55] Yamada H, Chounan R, Higashi Y, Kurihara N, Kido H (2004) Mitochondrial targeting sequence of the influenza A virus PB1-F2 protein and its function in mitochondria. FEBS Lett 578: 331–336. 15589841 10.1016/j.febslet.2004.11.017

[56] Jackson AC, Kammouni W, Fernyhough P (2011) Role of oxidative stress in rabies virus infection. Adv Virus Res 79: 127–138. 21601046 10.1016/B978-0-12-387040-7.00008-1

[57] Grek CL, Zhang J, Manevich Y, Townsend DM, Tew KD (2013) Causes and consequences of cysteine S-glutathionylation. J Biol Chem 288: 26497–26504. 23861399 10.1074/jbc.R113.461368 PMC3772197

[58] Prinarakis E, Chantzoura E, Thanos D, Spyrou G (2008) S-glutathionylation of IRF3 regulates IRF3-CBP interaction and activation of the IFN beta pathway. EMBO J 27: 865–875. 18309294 10.1038/emboj.2008.28 PMC2274937

[59] Quarato G, Scrima R, Agriesti F, Moradpour D, Capitanio N, et al (2013) Targeting mitochondria in the infection strategy of the hepatitis C virus. Int J Biochem Cell Biol 45: 156–166. 22710347 10.1016/j.biocel.2012.06.008

[60] Silva da Costa L, Pereira da Silva AP, Da Poian AT, El-Bacha T (2012) Mitochondrial bioenergetic alterations in mouse neuroblastoma cells infected with Sindbis virus: implications to viral replication and neuronal death. PLoS One 7: e33871. 22485151 10.1371/journal.pone.0033871 PMC3317446

[61] Cogliati S, Frezza C, Soriano ME, Varanita T, Quintana-Cabrera R, et al (2013) Mitochondrial cristae shape determines respiratory chain supercomplexes assembly and respiratory efficiency. Cell 155: 160–171. 24055366 10.1016/j.cell.2013.08.032 PMC3790458

[62] Koshiba T, Yasukawa K, Yanagi Y, Kawabata S (2011) Mitochondrial membrane potential is required for MAVS-mediated antiviral signaling. Sci Signal 4: ra7. 21285412 10.1126/scisignal.2001147

[63] Sasaki O, Yoshizumi T, Kuboyama M, Ishihara T, Suzuki E, et al (2013) A structural perspective of the MAVS-regulatory mechanism on the mitochondrial outer membrane using bioluminescence resonance energy transfer. Biochim Biophys Acta 1833: 1017–1027. 23337771 10.1016/j.bbamcr.2013.01.010

[64] Kim S, Kim HY, Lee S, Kim SW, Sohn S, et al (2007) Hepatitis B virus x protein induces perinuclear mitochondrial clustering in microtubule- and Dynein-dependent manners. J Virol 81: 1714–1726. 17151129 10.1128/JVI.01863-06 PMC1797565

[65] Nomura-Takigawa Y, Nagano-Fujii M, Deng L, Kitazawa S, Ishido S, et al (2006) Non-structural protein 4A of Hepatitis C virus accumulates on mitochondria and renders the cells prone to undergoing mitochondria-mediated apoptosis. J Gen Virol 87: 1935–1945. 16760395 10.1099/vir.0.81701-0

[66] El-Bacha T, Menezes MM, Azevedo e Silva MC, Sola-Penna M, Da Poian AT (2004) Mayaro virus infection alters glucose metabolism in cultured cells through activation of the enzyme 6-phosphofructo 1-kinase. Mol Cell Biochem 266: 191–198. 15646042 10.1023/b:mcbi.0000049154.17866.00

[67] Miller S, Krijnse-Locker J (2008) Modification of intracellular membrane structures for virus replication. Nat Rev Microbiol 6: 363–374. 18414501 10.1038/nrmicro1890 PMC7096853

[68] Nchoutmboube JA, Viktorova EG, Scott AJ, Ford LA, Pei Z, et al (2013) Increased long chain acyl-Coa synthetase activity and fatty acid import is linked to membrane synthesis for development of picornavirus replication organelles. PLoS Pathog 9: e1003401. 23762027 10.1371/journal.ppat.1003401 PMC3675155

[69] Parekh AB (2003) Mitochondrial regulation of intracellular Ca2+ signaling: more than just simple Ca2+ buffers. News Physiol Sci 18: 252–256. 14614159 10.1152/nips.01458.2003

[70] Hollander JM, Thapa D, Shepherd DL (2014) Physiological and Structural Differences in Spatially-Distinct Subpopulations of Cardiac Mitochondria: Influence of Pathologies. Am J Physiol Heart Circ Physiol. 10.1152/ajpheart.00747.2013 PMC4080170 24778166

[71] Kaarbo M, Ager-Wick E, Osenbroch PO, Kilander A, Skinnes R, et al (2011) Human cytomegalovirus infection increases mitochondrial biogenesis. Mitochondrion 11: 935–945. 21907833 10.1016/j.mito.2011.08.008

[72] Rojo G, Chamorro M, Salas ML, Vinuela E, Cuezva JM, et al (1998) Migration of mitochondria to viral assembly sites in African swine fever virus-infected cells. J Virol 72: 7583–7588. 9696857 10.1128/jvi.72.9.7583-7588.1998 PMC110008

[73] Shih SR, Stollar V, Lin JY, Chang SC, Chen GW, et al (2004) Identification of genes involved in the host response to enterovirus 71 infection. J Neurovirol 10: 293–304. 15385252 10.1080/13550280490499551

[74] Claus C, Schonefeld K, Hubner D, Chey S, Reibetanz U, et al (2013) Activity increase in respiratory chain complexes by rubella virus with marginal induction of oxidative stress. J Virol 87: 8481–8492. 23720730 10.1128/JVI.00533-13 PMC3719815

[75] Rossignol R, Faustin B, Rocher C, Malgat M, Mazat JP, et al (2003) Mitochondrial threshold effects. Biochem J 370: 751–762. 12467494 10.1042/BJ20021594 PMC1223225

[76] Chang SC, Lin JY, Lo LY, Li ML, Shih SR (2004) Diverse apoptotic pathways in enterovirus 71-infected cells. J Neurovirol 10: 338–349. 15765805 10.1080/13550280490521032

[77] Korytowski W, Basova LV, Pilat A, Kernstock RM, Girotti AW (2011) Permeabilization of the mitochondrial outer membrane by Bax/truncated Bid (tBid) proteins as sensitized by cardiolipin hydroperoxide translocation: mechanistic implications for the intrinsic pathway of oxidative apoptosis. J Biol Chem 286: 26334–26343. 21642428 10.1074/jbc.M110.188516 PMC3143596

[78] Harding HP, Calfon M, Urano F, Novoa I, Ron D (2002) Transcriptional and translational control in the Mammalian unfolded protein response. Annu Rev Cell Dev Biol 18: 575–599. 12142265 10.1146/annurev.cellbio.18.011402.160624

[79] Liu ZW, Zhu HT, Chen KL, Dong X, Wei J, et al (2013) Protein kinase RNA-like endoplasmic reticulum kinase (PERK) signaling pathway plays a major role in reactive oxygen species (ROS)-mediated endoplasmic reticulum stress-induced apoptosis in diabetic cardiomyopathy. Cardiovasc Diabetol 12: 158. 24180212 10.1186/1475-2840-12-158 PMC4176998

[80] Verfaillie T, Rubio N, Garg AD, Bultynck G, Rizzuto R, et al (2012) PERK is required at the ER-mitochondrial contact sites to convey apoptosis after ROS-based ER stress. Cell Death Differ 19: 1880–1891. 22705852 10.1038/cdd.2012.74 PMC3469056

[81] Joshi M, Kulkarni A, Pal JK (2013) Small molecule modulators of eukaryotic initiation factor 2alpha kinases, the key regulators of protein synthesis. Biochimie 95: 1980–1990. 23939221 10.1016/j.biochi.2013.07.030

[82] Donnelly N, Gorman AM, Gupta S, Samali A (2013) The eIF2alpha kinases: their structures and functions. Cell Mol Life Sci 70: 3493–3511. 23354059 10.1007/s00018-012-1252-6 PMC11113696

[83] Ito T, Yang M, May WS (1999) RAX, a cellular activator for double-stranded RNA-dependent protein kinase during stress signaling. J Biol Chem 274: 15427–15432. 10336432 10.1074/jbc.274.22.15427

